# A Conceptual Model of Tactile Processing across Body Features of Size, Shape, Side, and Spatial Location

**DOI:** 10.3389/fpsyg.2019.00291

**Published:** 2019-02-26

**Authors:** Luigi Tamè, Elena Azañón, Matthew R. Longo

**Affiliations:** ^1^ Department of Psychological Sciences, Birkbeck University of London, London, United Kingdom; ^2^ School of Psychology, University of Kent, Canterbury, United Kingdom; ^3^ Institute of Psychology, Otto von Guericke University Magdeburg, Magdeburg, Germany; ^4^ Center for Behavioral Brain Sciences, Magdeburg, Germany; ^5^ Department of Behavioral Neurology, Leibniz Institute for Neurobiology, Magdeburg, Germany

**Keywords:** somatosensory processing, space, body representation, laterality, body shape

## Abstract

The processing of touch depends of multiple factors, such as the properties of the skin and type of receptors stimulated, as well as features related to the actual configuration and shape of the body itself. A large body of research has focused on the effect that the nature of the stimuli has on tactile processing. Less research, however, has focused on features beyond the nature of the touch. In this review, we focus on some features related to the body that have been investigated for less time and in a more fragmented way. These include the symmetrical quality of the two sides of the body, the postural configuration of the body, as well as the size and shape of different body parts. We will describe what we consider three key aspects: (1) how and at which stages tactile information is integrated between different parts and sides of the body; (2) how tactile signals are integrated with online and stored postural configurations of the body, regarded as priors; (3) and how tactile signals are integrated with representations of body size and shape. Here, we describe how these different body dimensions affect integration of tactile information as well as guide motor behavior by integrating them in a single model of tactile processing. We review a wide range of neuropsychological, neuroimaging, and neurophysiological data and suggest a revised model of tactile integration on the basis of the one proposed previously by Longo et al.

## Introduction

There are multiple factors that determine how tactile stimuli on our body are processed to produce coherent tactile experiences and guide motor behavior. A large body of research over the past decades has focused on the effects that direct changes in the nature of the stimuli, such as texture (Johnson and Hsiao, 1992), inter-stimuli delays (Craig, 1983), duration (Gescheider and Migel, 1995), frequency (Gescheider et al., 2002), and intensity (Craig, 1974), have on the somatosensory response. Less research, however, has focused on body features that critically affect tactile processing beyond the physical parameters of the touch. These features include the size, shape, and spatial configuration of the body part stimulated, as well as the integration across different parts and sides of the body. In this review, we will focus on these features and describe: (1) how and at which stages tactile information is integrated between different parts and sides of the body; (2) how tactile signals are integrated with online and stored postural configurations of the body and/or locations in space; and (3) how tactile signals are integrated with stored models of body size and shape. We will describe how these different body dimensions affect integration of tactile information to produce a coherent representation of touch and perception of the body as an integrated whole.

Several years ago, two of us proposed a model of somatosensory information processing (Longo et al., 2010). The central premise of this model was that the processing of tactile information goes beyond primary *somatosensation,* by integrating immediate sensory signals with stored representations of the body. This type of higher order somatosensory processing, or somatoperception, contributes to somatic perceptual constancy, providing a coherent tactile percept on the body and contributing to the formation of the bodily self. In this model, we described how information from the body surface is remapped into an egocentric reference frame, how information about the shape and size of the body interacts with tactile processing, and the role that exteroceptive (i.e., perception of objects in the external world through their contact with the body) and interoceptive perception (i.e., percepts about the nature and state of the body itself) has in tactile perception. As described in the original papers (Longo et al., 2010, 2015b), the model is consistent with a wide range of neuropsychological, neuroimaging, and neurophysiological data.

At the core of this model is the claim that many aspects of higher level perceptions about somatosensory stimuli require that sensory signals be integrated with stored representations about the body itself. Specifically, Longo et al. (2010) postulated three distinct mental body representations: the superficial schema, the postural schema, and the body model. The superficial and postural schemas were first postulated by Head and Holmes (1911) on the basis of their studies of brain-damaged patients. One group of patients could detect that they had been touched, but could not perceive *where* on their skin the touch had been applied. Another group of patients could perceive the location of touch, but could not tell where their affected limb was in space when they could not see it. Head and Holmes postulated the existence of the superficial and postural schemas to account for the impairments of these two groups of patients, respectively. In the model of Longo et al. (2010), the superficial schema is described as a mapping between locations within primary somatotopic maps and locations on the skin surface. The postural schema, in contrast, is a more dynamic representation of current body posture (i.e., joint angles), incorporating both afferent proprioceptive signals and efferent copies of motor commands. Finally, Longo et al. (2010) proposed a third representation of the metric properties (i.e., size and shape) of the body, which they called the body model.

In this paper, we address some further factors, which were not addressed by the model of Longo et al. (2010). A first aspect is the fact that the body is bilaterally symmetric, with homologous locations on the right and left sides of the body. A second aspect is the use of prior locations and stored postural configurations of the body when localizing touch. Here, we attempt to integrate laterality into their model as well as the use of prior information, with the aim of describing how touch is processed given the duality of the body (i.e., left and right side) and brain structures (i.e., left and right hemispheres), which goes hand in hand with the perception of the body as a single unit. Finally, we review recent advances in understanding the integration of touch and higher level representations of body size and shape, an issue at the core of the model.

## Integration of Tactile Information between the Two Sides of the Body

Coordination between the two hemispheres is paramount for perception and motor control of the body. Indeed, early processing of tactile signals occurring on the two sides of the body is critical to perform appropriate goal-directed bimanual motor tasks. This notion seems to clash with the classical view that unilateral tactile stimuli are represented only in the contralateral primary somatosensory cortex (SI) (Penfield and Boldrey, 1937; Nelson and Chen, 2008). Indeed, the somatosensory and motor systems require continuous and sudden switches between lateralized and joint interhemispheric processing. Such processing includes the execution of simple actions, as well as more complex goal-directed motor behaviors. The stage of tactile sensory processing at which the interhemispheric transfer of tactile information occurs is still matter of debate (Allison, et al., 1989; Kanno, et al., 2003; Hlushchuk and Hari, 2006; Sutherland, 2006; Tommerdahl et al., 2006; Jung et al., 2012; Tamè, et al., 2016). In this section, we will describe some recent evidence in humans suggesting an early interhemispheric integration of tactile signals between the two hemispheres, possibly serving the execution of appropriate motor behavior.

### Behavioral Evidence of Tactile Interhemispheric Communication in Healthy Subjects

The first stage of bilateral integration of tactile information, at cortical level, is generally thought to occur in brain areas beyond the primary somatosensory cortex (SI; Eickhoff et al., 2010); however, recent evidence have shown that SI contributes to such a processing (Kanno et al., 2004; Tan, et al., 2004; Tommerdahl et al., 2006; Tamè et al., 2012). In macaques, bilateral receptive fields have been described as early as somatosensory area 2 (Iwamura et al., 1994, 2002), an area considered to be the homologue of Brodmann area 2 (BA 2) of human primary somatosensory cortex. Furthermore, interhemispheric interactions have been observed for stimuli presented to both paws, even in the core area of SI (area 3b) of owl monkeys (Lipton et al., 2006; Reed et al., 2010, 2011).

**Figure 1:**
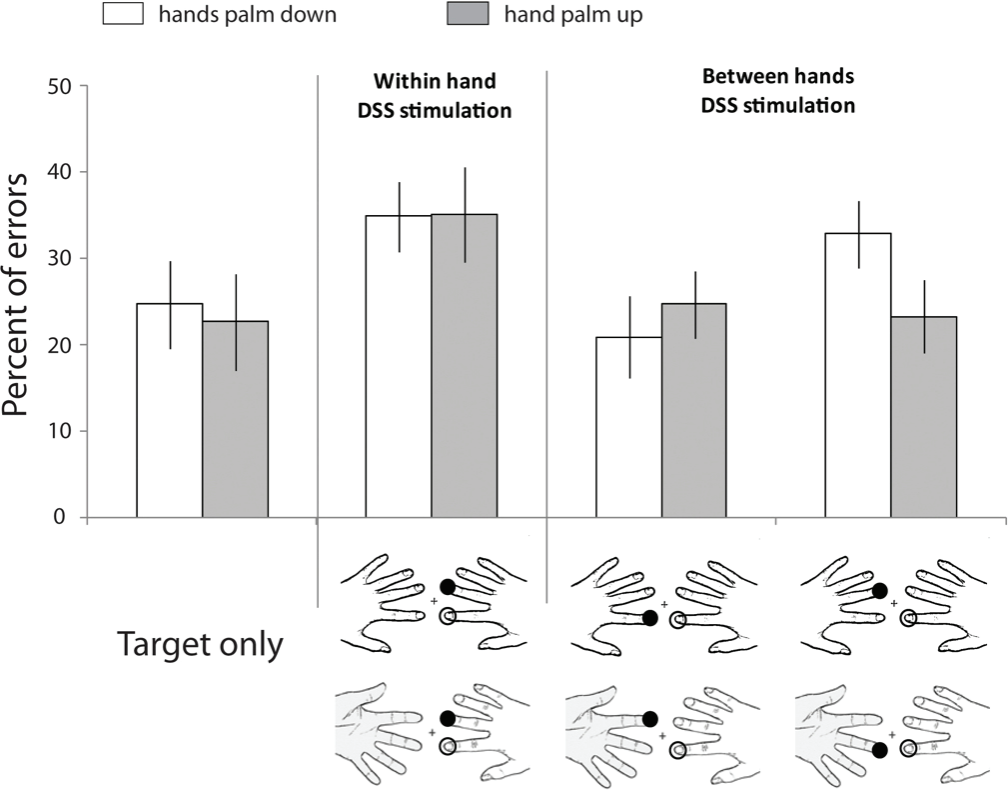
Spatial coding of touch at the fingers. Data retrieved from Tamè et al. (2011) study in which participants performed a speeded go-no-go task to indicate whether the target finger had been stimulated or not. Across conditions, the target finger was presented alone or concurrently with a masker (double simultaneous stimulation, DSS) on another finger (i.e., other finger of the same hand, homologous finger of the opposite hand, non-homologous finger of the opposite hand). Moreover, in different blocks, participants assumed different postures (i.e., hands palm down or hand palm up). Unfilled circles: Stimulation at the target finger; filled black circles: stimulation at the non-target finger. Bar plots show percent errors as a function of stimulation condition and hands’ posture. Error bars represent the standard error of the mean (±SEM). Adapted from Tamè et al. (2011). © 2011 by Elsevier. Permission for the use of the image has been obtained from the Elsevier.

In humans, there is growing evidence about how and when this exchange of tactile information between the two hemispheres is likely to occur (Tamè et al., 2016). For instance, Tamè and colleagues developed a paradigm of double simultaneous tactile stimulation (DSS; Tamè et al., 2011, 2013). In this study, participants were instructed to detect the presence of a tactile stimulus on a target finger. Depending on the condition, the target finger was stimulated in isolation or concurrently with another finger (i.e., masker finger). The masker was a stimulus on a finger of the same or a different hand (i.e., index and middle fingers of both hands). In accordance with previous literature, results showed that when a masker was present there was an interference effect regardless of the stimulated hand. However, critically the amount of interference varied as a function of the stimulated finger rather than the hand (i.e., which hemibody was touched; see Figure 1). The same interference was present when the non-homologous finger, with respect to the target, was the masker regardless of the hand. By contrast, such interference was significantly reduced when the masker was the homologous finger of the other hand. Therefore, the information is differently processed for homologous body parts (compared to non-homologous), as if they were coming from the same side of the body (for similar evidence on fingers homology interactions across side using a different paradigm, see Rusconi et al., 2014). This somatotopic organization provides indirect evidence that SI is involved in the side integration processing of touch. Such integration is altered when the spatial relationships between the hands/fingers change (Tamè et al., 2011). These last findings are in agreement with those reported by Haggard et al. (2006), who showed that under tactile stimulation, identification of the hand is affected by changes in hand posture, whereas this is not the case for the identification of the finger. Specifically, these authors suggested that tactile detection and finger identification occur at a somatotopic representational level, whereas hand identification occurs at a higher level in which postural information are taken into account. The role of the postural configuration in tactile processing will be widely discussed in the next section.

### Neuroimaging Evidence of Tactile Interhemispheric Communication in Healthy Subjects

Furthermore, using functional magnetic resonance imaging (fMRI), Tamè et al. (2012) identified the neural bases of bilateral integration of touch on homologous and non-homologous fingers of the two hands. In particular, Tamè and colleagues used an fMRI tactile adaptation paradigm in which pairs of vibrotactile stimuli were delivered on the left and right index and middle fingers. The adaptation paradigm relies on the reduced response of certain neurons that results from the repeated presentation of a specific feature to which these neurons are selective. On this basis, Tamè et al. (2012) hypothesized that if there are neurons that have finger-specific selectivity (i.e., index and middle fingers) a greater adaptation should emerge when the index finger (i.e., same finger) is stimulated twice compared to when different fingers are stimulated (i.e., index and middle fingers). They expected that such a pattern should emerge in SI, which is known to hold somatotopic representations. Critically, if SI is also capable of integrating stimuli that come from the two sides of the body, such a pattern should be present regardless of the side of stimulation (i.e., fingers of the left and right hand). Tamè et al. (2012) found that BOLD response was indeed greatly reduced in SI, as well as in SII, when the same finger was stimulated twice (index-index) compared to when different fingers were stimulated (middle-index), both when stimuli were delivered on the same and different hands. This result proved that SI can integrate tactile stimuli coming from the two sides of the body. The most likely subarea(s) of SI responsible for mediating such a processing can be identified as areas BA1 and BA2. Indeed, using the SPM (Statistical Parametric Mapping) anatomy toolbox, Tamè et al., 2012 identified the origin of their BOLD response in such areas. This is also compatible with studies on monkeys which showed the presence of bilateral receptive fields in area 2 (Iwamura et al., 2002). In order to overcome the limited temporal resolution of fMRI, in a subsequent study, Tamè and colleagues used a magnetoencephalography (MEG) adaptation paradigm to investigate whether the integration of bilateral tactile stimuli in SI occurred at early or late stages of tactile processing (Tamè et al., 2015). The results showed that when tactile stimuli were delivered on different hands, neural responses were somatotopically constrained, being smaller for stimulation of homologous than non-homologous fingers. Importantly, neural responses of the tactile stimuli of the two sides of the body interacted in SI at short delays (i.e., 25 ms). This is most likely due to the fact that the temporal integration window in SI is short (Mauguière et al., 1997) and long in SII (Wühle et al., 2011), suggesting that selective interaction for short delays is likely to occur within SI, rather than deriving from modulatory effects from higher level brain areas. Therefore, this pattern of results provides substantial evidence that integration of bilateral tactile stimuli on the hands cannot solely derive from higher stages of the tactile representation processing (i.e., SII and beyond) as previously suggested by other reports (Jung et al., 2012; Chung et al., 2014). The discrepancy between these results and some previous studies can be ascribed to different factors. A first possibility is that Tamè et al.’s (2015) adaptation approach has a greater sensitivity to detect changes in the neural activity in the somatosensory cortex under bilateral stimulation (Tamè et al., 2016). Indeed, this is not a trivial problem given the overwhelming response generated in the contralateral hemisphere following unilateral tactile stimulation. Another possibility, not mutually exclusive with the one just described, is the different type and locus of stimulation they used in their study compared to other works. Tamè et al. (2015) used a mechanical piezo tactile stimulator (i.e., a matrix of 2 × 5 rods; 1 mm in diameter) applied on the first phalange of the index and middle fingers for 12 ms. Instead, Cheng et al. (2014) stimulated the right index finger using a band-type MR-compatible device that pressed the whole ventral skin surface of the finger for 3 seconds, a rather long stimulation compared to Tamè et al., 2015. Moreover, Jung et al. (2012) used constant-current square-wave pulse stimulation with a very short duration (i.e., 0.2 ms), though they stimulated the median nerve of both hands at the level of the wrist, rather than the fingers as Tamè et al. (2015) did.

Overall, this result suggests that tactile stimuli from the two sides of the body (i.e., fingers) interact at an early stage of the tactile representation processing in the primary somatosensory cortex, most likely through transcallosal pathways which connect SI in the two hemispheres (see also the graphical representation of the transcallosal pathways model, Figure 3 in Tamè et al., 2016).

### Sensorimotor Interhemispheric Communication in Healthy Subjects

A recent study by Tamè and Longo (2015) provided behavioral evidence of the role of topographical organization of callosal connections in the integration of sensorimotor (i.e., touch) stimuli across the two sides of the body. Using a classical behavioral paradigm to quantify sensorimotor transfer between hemispheres, i.e., the Poffenberger paradigm (Poffenberger, 1912), the study revealed a modulation of the sensorimotor interhemispheric integration time as a function of the body part stimulated. The Poffenberger paradigm relies on the logic that sensorimotor information is integrated and processed within the same hemisphere when a motor effector and the sensory signal are on the same side of the body (uncrossed). This behavioral paradigm is based on the fact that people respond faster (lower reaction times: RTs) when sensory stimuli are presented in the hemifield (for visual or auditory stimuli) or hemibody (for tactile stimuli) ipsilateral to the hand used to respond (i.e., sensory stimulus and motor response occur in the same hemisphere: uncrossed) than contralateral (sensory stimulus and motor response occur in different hemispheres: i.e., crossed). Poffenberger proposed that the time required for signals to transfer between the two cerebral hemispheres is reflected by the crossed-uncrossed difference (CUD) (Poffenberger, 1912; Marzi, 1999). By contrast, if sensory input and motor effector belong to different sides of the body, the information has to be integrated across hemispheres (crossed). In their study, the authors showed that the crossed-uncrossed difference in processing time was larger on the finger (2.6 ms) and forearm (1.8 ms) than on the forehead (0.9 ms; Tamè and Longo, 2015). The callosal connections and density of bilateral receptive fields (RFs) are consistent with such temporal difference. Indeed, it has been shown that regions that represent the periphery of body have less dense callosal connections compared to regions that represent the center (Pandya and Vignolo, 1969; Caminiti and Sbriccoli, 1985; Iwamura et al., 2001). This result suggests that the interhemispheric integration of sensorimotor stimuli, at least in the tactile domain, varies as a function of the strength of callosal connections of the body parts (Tamè and Longo, 2015). Interestingly, the cost that is paid when processing a stimulus that is on the contralateral side with respect to the effector can be vanished when touch is delivered on a seen hand. Therefore, the interhemispheric integration of tactile-motor responses can be improved by vision of the body (cf. Tamè et al., 2017a). A question that is interesting to ask is, which are the possible mechanisms that can account for this result? A first possibility is that participant’s performance is enhanced by improving their motor performance when seeing the hand. Indeed, it has been shown that when participants have to perform a goal-directed action, seeing their own hand starting point enhances their performance in the motor task (Prablanc et al., 1979; Rossetti et al., 1994; Blanchard et al., 2013). Similarly, another study has shown that manual responses are primed by the vision of the participant’s own hand (Longo and Haggard, 2009). A second possibility is that some attentional mechanisms are mediating such effect. Indeed, when participants see their own hand, a facilitatory effect occurs, which improves the processing of spatial tactile information selection on the body and/or attenuates the conflictual response coding between the stimulus and effector when they belonged to different body sides (Pierson et al., 1991). Note that these two cases may not be mutually exclusive. The neural substrate of such a processing is unclear; therefore, future studies should try to provide empirical evidence to define such mechanisms. Having said that, however, we know that when non-informative vision of the body is present participants give faster responses to touch compared to when vision of the body is absent, a phenomenon named “visual enhancement of touch” (VET; Tipper et al., 1998; Kennett et al., 2001). The neural correlates of such effect are thought to derive from a multisensory modulatory effect from the parietal cortex (Ro et al., 2004) where there are bimodal neurons (Graziano et al., 1994) that preactivate the somatosensory cortex improving tactile performance. Alternatively, in the study of Tamè et al. (2017a), the primary somatosensory cortex may have processed such information through a coupling with the visual areas. Indeed, it has been suggested that the “low-level” sensory areas may be multisensory in nature (Ghazanfar and Schroeder, 2006; Macaluso, 2006; Bruno and Pavani, 2018; Convento et al., 2018; Holmes and Tamè, 2018). However, the effect reported by Tamè and colleagues (Tamè et al., 2017a) cannot be solely explained by such a perceptual mechanism, given that they found faster responses to touch when vision of the body was present only in the contralateral hemisphere, i.e., stimulus and effector on different sides of the body, but not in the ipsilateral. Therefore, further studies are needed to clarify the mechanisms as well as the neural correlates of the improvement of interhemispheric integration of tactile-motor responses by vision of the body possibly through the integration of the perceptual and motor perspectives.

Moreover, other research has demonstrated that task demands can modulate tactile perception and processing as well as brain areas involved (e.g., Pritchett et al., 2012; Romo et al., 2012; Tamè and Holmes, 2016). In particular, relevant to the present context, finger-specificity interactions for tactile stimuli delivered on the two sides of the body are present only when complex tactile tasks (i.e., tactile detection in a go-no-go context, tactile localization, and discrimination) have to be accomplished (e.g., Tamè et al., 2011, 2017c; Dempsey-Jones et al., 2015), but not when simpler tactile tasks (i.e., tactile detection in a two-intervals force choice design) have to be solved (e.g., Tamè et al., 2014). Indeed, in the latter case, Tamè et al. (2014) showed that tactile interference is the same regardless of the stimulated fingers of the two hands (Tamè et al., 2014). Therefore, the topographic organization in the bilateral interaction is modulated by the specific task demands (Tamè et al., 2016).

### Neuropsychological Evidence of Tactile and Motor Interhemispheric Communication

Sensory interhemispheric communication has also been studied in brain-damaged patients. A typical neuropsychological example of bilateral integration is patients with tactile extinction. Such individuals are perfectly capable of detecting a single tactile stimulus on one or the other side of the body. However, when two tactile stimuli are delivered simultaneously on the two body sides, patient fail to report the contralateral stimulus with respect to the locus of the lesion (Bender, 1945). Other neuropsychological examples are provided by mislocalization or reduplication phenomena. Mislocalization of touch across body sides has been termed *allochiria* (Obersteiner, 1881), whereas reduplication has been termed *synchiria* (Jones, 1908). Arm amputees and brain-damaged patients with hemiparesis and hemisensory loss are cases in which *allochiria* has been described (Bisiach and Berti, 1995) and in which these individuals can report contralateral referral of tactile sensations to the phantom body part (Ramachandran et al., 1995) or to the hand rendered anesthetic by stroke (Sathian, 2000).

Medina and Rapp (2008) described a case of tactile *synchiria* in which an individual who suffered from a left frontoparietal damage experienced bilateral tactile sensations after unilateral stimulation. The authors ascribed this effect primarily to a deficit in the inhibitory mechanisms that, in healthy individuals, naturally suppress the bilateral percept. This interesting interpretation would support the notion that unilateral tactile stimulation is capable to produce signals in both hemispheres.

Other conditions in which tactile referral to other body parts emerges are provided by patients who show mirror movements across homologous body parts. For instance, Farmer et al. (1990) studied a patient who suffered from the Klippel-Feil syndrome, a skeletal abnormality that is typically associated with mirror movements of the hand muscles (Bauman, 1932), in which voluntary activation of a muscle is replicated by an identical involuntary movement in the homologous muscle of the opposite hand. Interestingly, the authors found that unilateral electrical stimulation of the index finger produces an excitatory response in the stimulated side as well as a bilateral excitatory response approximately equal size and latency, whereas in the healthy subjects such a response was only present in the stimulated side (Farmer et al., 1990). Compatible with the idea of similarity between homologous parts of the two sides of the body, a recent study investigating the contribution of proprioceptive signals from the two sides of the body in the control of joint movements suggests the existence of a control programme that is common and uses proprioceptive information from the same joints of the two sides of the body (Han et al., 2013).

Based on these findings, Tamè et al. (2016) suggested that tactile information is integrated through transcallosal pathways connecting SI of the two hemispheres. Here, we aim to integrate this proposal into the model of somatoperceptual information processing developed by Longo et al. (2010; 2015b). In particular, we suggest that afferent tactile inputs from the two sides of the body reach Brodmann (BA) areas 3a and 3b of the contralateral primary somatosensory cortex, then continue to areas 1 and 2 – which also receive direct inputs from the thalamus – where the signals between the two sides of the body are integrated. At this point, tactile laterality is communicated to other brain areas within (i.e., 3a and 3b) and beyond (parietal areas as well as motor and premotor cortices) SI. Such integration process can have an important advantage. Indeed, it would be inefficient to maintain double representations of each body part along the whole tactile processing pathway, given that the structure of the body is homologous on either side of the body midline. Therefore, at higher level representation stages, beyond *somatosensation* using Longo et al.’s (2010) nomenclature, tactile inputs are processed using a single body model, which does not distinguish between the left and right body side.

The presence of a single body representation, for both sides of the body, is further suggested by neuropsychological evidence in patients suffering from left parietal lesions. For instance, it has been proposed that the *body structural representation* (BSR) is a critical component in mediating the knowledge about the spatial configuration of bodies. This notion relies on the fact that damage of such a representation results in conditions such as autotopagnosia (Ogden, 1985; Sirigu et al., 1991) and finger agnosia (Kinsbourne and Warrington, 1962). Studies of neurological patients (Schwoebel and Coslett, 2005) and healthy adults (Felician et al., 2004; Corradi-Dell’Acqua et al., 2009; Rusconi et al., 2014) provide evidence that the bilateral parietal cortex may mediate the structural representations of the body. A study by Rusconi and colleagues, using a bi-manual version of the in-between task (i.e., participants estimate the number of unstimulated fingers between two touched fingers), suggests that the left and right posterior parietal cortices contribute to the on-line sensorimotor representations (Pisella et al., 2000). Instead, they suggest that the connections between the left anteromedial inferior parietal lobe (a-mIPL) and the precuneus (PCN) provide the core substrate of an explicit bilateral BSR for the fingers that when disrupted can produce the typical symptoms of finger agnosia (Rusconi et al., 2014). Therefore, this study supports the notion of the presence of a single body model as a lateralized neural structure provides information about the representation of the body parts in space relative to each other that applies to the two sides of the body. Similarly, patients who suffer from *synchiria* are not able to distinguish anymore which is the side from where the tactile input is coming from, given that they perceive the sensation as occurring on both sides (Jones, 1908).

Furthermore, the study by Han et al. (2013), which we described above, may suggest that a similar integration flow is occurring also for the proprioceptive signals, though further evidence is needed to assess it. Indeed, proprioceptive signals for the control of joint movements may be controlled by a common programme that is the same for the left and right sides of the body. Such a possibility is compatible with the idea that tactile inputs are processed using a single body model, which does not distinguish between the two sides of the body.

**Figure 2:**
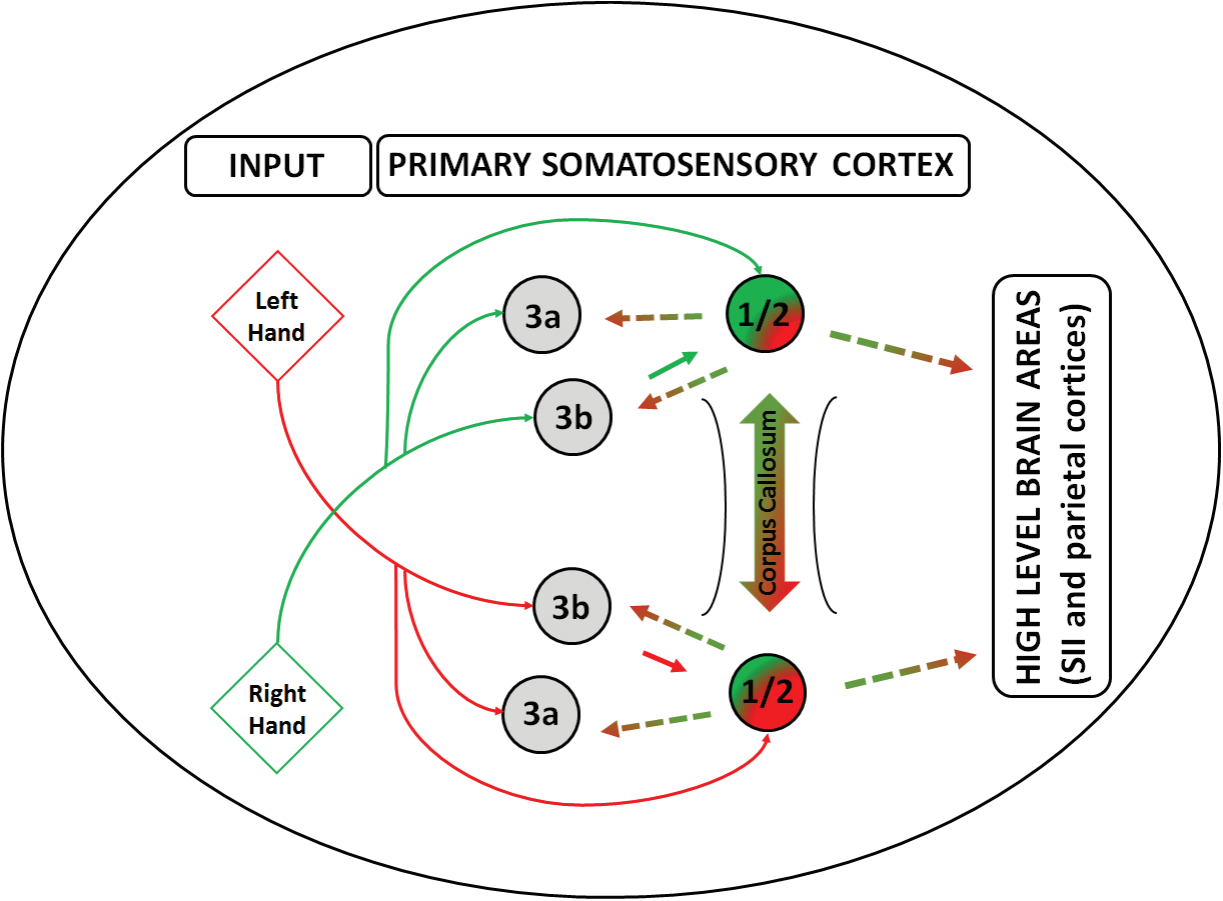
Side integration model. A graphical model of tactile laterality information processing – i.e., Side Integration Model, highlighting the role of areas 1 and 2 in the primary somatosensory cortex in the integration of the lateralized tactile inputs from the two sides of the body. Red lines depict the primarily pathways of information flow coming from the left body side from tactile and proprioceptive afference, whereas green lines depict information coming from the right body side. Gradient line depicts the integration of the inputs from the two sides of the body through the corpus callosum, whereas the dashed lines depict the information flow including the body laterality towards the other areas within the primary somatosensory cortex and beyond. Inputs are depicted as diamond shapes and cortical brain areas as circles.

Overall, the psychophysical, neurophysiological, neuroimaging, and neuropsychological evidence we described suggest that integration of the tactile signal between the two sides of the body – i.e., hands – is likely to occur at early stages of the tactile representation processing within the primary somatosensory cortex as depicted in Figure 2 (for an extensive review on this topic, see Tamè et al., 2016). Therefore, the afferent flow of tactile information from the thalamus reaches BA areas 3a and 3b of SI of the contralateral hemisphere with respect to the locus of stimulation who themselves project to areas 1 and 2 – which also have direct inflow of information from the thalamus. We propose that the side integration occurs in areas 1/2 of SI through transcallosal connections as shown by the neuroimaging studies in humans we described (Tamè et al., 2012, 2015; for a review see Tamè et al., 2016). Following this process, information about tactile laterality is communicated to other brain areas within SI (i.e., 3a, 3b), parietal areas, as well as the motor and premotor cortices (Sutherland, 2006). We do not have specific prediction about the nature of such a signal, i.e., excitatory or inhibitory, which most likely depends on the specific task demands. Future studies should focus on trying to provide further empirical evidence that can possibly support/rectify or reject this hypothesis. We believe that a sensitive approach to pursue this goal can be to perform a series of tactile tasks with different levels of complexity that involve bilateral tactile stimulation of the body as well as require side-dependent or independent representation of the body. Ideally, such approach should be performed in combination with the state-of-the-art neuroimaging techniques such as, for instance, fMRI (where in the brain this is occurring), EEG (when is occurring), and TMS.

## Integration of Tactile Information with Posture

The previous section has dealt with the integration across body sides, explicitly neglecting the role that posture has on tactile processing. However, even in tasks such as the ones reported so far, in which the goal is to report the exact finger that has been stimulated, proprioceptive information would still play a fundamental role. This is so, as localizing touch on a body surface is not by itself sufficient to interact with the environment (Driver and Spence, 1998). As we move, our bodies and limbs change position, and the relative location of each touch varies with respect to the body and other objects in the environment. It is because of this countless combination of tactile and proprioceptive signals, each indicating different locations in external space, that the brain needs to consider posture when processing touch. This integration allows representing touch beyond skin space, i.e., in an external reference frame, making it available for goal-directed actions (Driver and Spence, 1998; Yamamoto and Kitazawa, 2001). There is now a consensus in the literature that this integrative process of tactile remapping occurs by default, weighting each reference frame accordingly to task demands, even in situations where postural integration is unnecessary (Azañón and Soto-Faraco, 2008a; Azañón et al., 2010a; Badde et al., 2015; Heed et al., 2015).

In the present section, we will focus on this integration and describe evidence suggesting not only the integration of touch and online proprioceptive signals but also between touch and *a priori* information regarding specific locations in space (i.e., spatial priors) and/or canonical postural representations (i.e., prototypical postural configurations). These prior configurations or locations in space might enable faster motor responses to spatial locations where the occurrence of touch is more probable, allowing faster integration with other modalities, for instance, to avoid threating stimuli.

### The Role of Vision and Development in Tactile Spatial Perception

Studies of children provide evidence that the process of tactile remapping is acquired during development, probably through active interaction with the environment (Bremner et al., 2008a). Tactile remapping develops with age (Bremner et al., 2008b; Pagel et al., 2009; Begum et al., 2014; Rigato et al., 2014), it is not present in infants younger than 6–10 months (Bremner et al., 2008b; Rigato et al., 2014; Begum Ali et al., 2015), and it has been associated to the ability to perform the first reaches to objects across the body midline, which suggest a tight relation with experience (Bremner et al., 2008a; Rigato et al., 2014). Furthermore, studies of the congenitally blind provide further support of the role of early visual experience in the processing of tactile stimuli later in life (Röder et al., 2004). For instance, congenitally blind individuals, who have never experienced visual input, do not show a detriment in tactile localization performance when the hands are crossed as compared to uncrossed (Röder et al., 2004; Collignon et al., 2009). This is not the case, however, for sighted participants or people who have become blind later in life, even after many years of having lost sight: performance with hands crossed is largely impaired as compared to uncrossed, even in situations where posture is irrelevant (Röder et al., 2004). This suggests that extensive visual experience during the first years of life leads to a default encoding of touch in terms of external space, even in cases where taking posture into account is detrimental. In support to this idea, the deprivation of visual input during the first years of life, by congenital dense bilateral cataracts in humans, hinders the normal development of a default remapping of touch in external space (Ley et al., 2013; Azañón et al., 2018).

Through acting in the world, sighted individuals are exposed to continuous sensorimotor contingencies across signals from the various modalities. Tactile spatial perception, thus, might therefore emerge as the repeatedly experienced correlation of specific activity of skin receptors with proprioceptive and visual information about limb position and the object touching the skin (Heed et al., 2015). This idea comes across clearly in Nissen et al. (1951), where a chimpanzee was raised from birth with pads covering arms and legs. These pads allowed the chimpanzee to move but prevented climbing and any manipulative behavior. The lack of opportunity for manipulation and for association of visual with tactile-kinesthetic sensations compromised to large extent basic tactile orienting responses later in life, such as orienting the head to the location of single touches presented to either hand. This suggests a large degree of impairment in basic tactile spatial processing after sensorimotor deprivation.

### Spatial Priors and/or Canonical Postural Representations

Under a framework in which tactile spatial perception emerges through active exploration with the environment, it is plausible that with experience, initially uncorrelated distributions of locations in space across tactile, proprioceptive, and visual signals become correlated during development. For instance, given the morphology and physical constraints of the arm, touches on the right hand would occur more often on the right side and around the center of the body, with respect to the body midline. This frequent co-occurrence of sensory signals in particular locations of space might promote the emergence of visual *spatial priors*, serving as reference points for localization of tactile events, analogous to the use of spatial prototypes, or Bayesian priors in other forms of spatial representation (Huttenlocher et al., 1991; Körding and Wolpert, 2004). Similarly, frequent occurrence of touch while adopting particular body configurations might promote the emergence of proprioceptive *canonical postures* (i.e., prototypical postural configurations).

Note that spatial priors and canonical proprioceptive configurations could produce similar behavioral effects but correspond to two separate concepts. Spatial priors, as defined in this review, do not require stored proprioceptive information, but stored representations about the most plausible locations of touch in visual space (e.g., touches on the right hand would occur more often on the right side). To our knowledge, this is the first time, the concept of spatial prior, as defined in visual space, has been linked to tactile remapping. The concept of canonical posture, more widespread than the concept of spatial prior in the literature of remapping (Yamamoto and Kitazawa, 2001; Azañón and Soto-Faraco, 2008a; Bremner et al., 2008a,b; Longo et al., 2010), assumes the existence of stored proprioceptive representations, which contain the most plausible body configurations for a given touch (i.e., for a touch on the hand, the canonical configuration assumes uncrossed arms).

**Figure 3:**
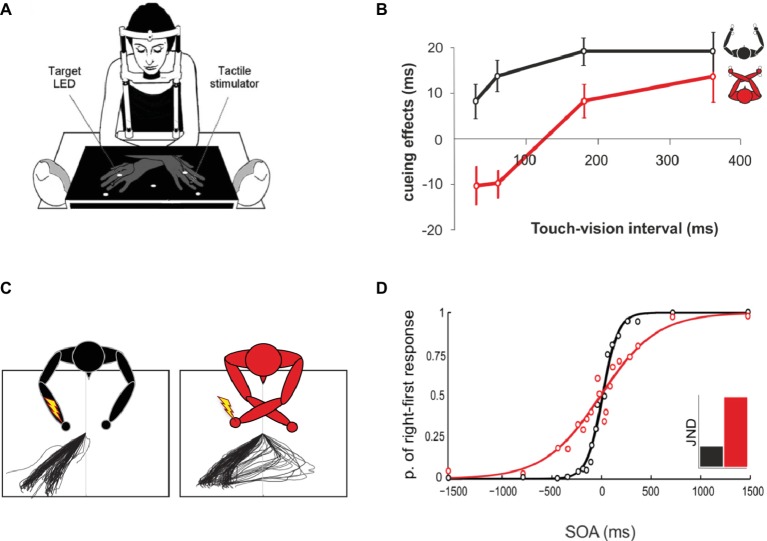
The use of prior information in tactile spatial localization. **(A)** Data retrieved from Azañón and Soto-Faraco (2008a) study, in which participants were asked to judge as quickly as possible the position of a light flash in the vertical dimension (top-bottom), irrespective of the side of presentation and location of an irrelevant tactile cue. **(B)** At short cue-target intervals (<60), with arms crossed (red line), targets were faster in opposite cue-target side trials than in same side trials. The pattern reversed after cue-target intervals of about 200 ms, so that tactile cues produced a facilitation of targets presented at the same external location. No differences across intervals were found with uncrossed hands (black line). **(C)** Data were retrieved from Overvliet et al.’s (2011) study, where participants were asked to direct saccades to a tactile stimulus at the ring finger of one of the two hands, which could be either crossed or uncrossed. Saccades to tactile stimuli when the hands were crossed (right-most panel) were sometimes initiated to the wrong direction and then corrected in-flight, resulting in a turn-around saccade. Adapted from Overvliet et al. (2011). © 2011 by Elsevier. Permission for the use of the image has been obtained from the Elsevier. **(D)** Figure modified from Heed and Azañón (2014). Typical single participant results of uncrossed and crossed hands temporal order judgment. In this task, two touches are presented at different stimulus onset asynchronies (SOA), and participants are required to move the finger that has been stimulated first, with no time restrain. With uncrossed hands (black line), the psychophysical curve is steeper than with crossed hands (red line), indicating an advantage in performance for the uncrossed posture. The inset illustrates the just noticeable difference (JND) for uncrossed and crossed postures.

The existence of spatial priors is clear in vision. For instance, it has been shown that memories of spatial locations are biased towards particular locations of space in a highly stereotyped manner and across individuals. For instance, when recalling the location of a dot inside a circle, participants’ responses are biased towards the centroids of each quadrant (Huttenlocher et al., 1991, 2004). A widespread assumption from this type of result is that by integrating the memory for the actual stimulus with categorical information about where stimuli are expected to be, perceptual accuracy can be increased, though at the expense of introducing systematic bias (Cheng et al., 2007). Similarly, spatial priors in touch might provide accurate and faster tactile localization performance, pulling in nearby stimuli (as shown for visual priors), but also increase errors when large mismatches occur between the spatial prior (defined in visual space) and online tactile-proprioceptive signals. This could explain why crossing the hands produce more tactile localization errors than when the hands are at its anatomical and, therefore, expected location (see Figure 3D; Yamamoto and Kitazawa, 2001; Shore et al., 2002).

In light with the idea that frequent co-occurrence of sensory signals can lead to the establishment of priors, Azañón et al. (2015) have recently shown that repetition of touch in the same crossed posture, even if unattended, can lead to an improvement in tactile localization, which increases with respect to the number of preceding trials. These results hence confirm that recent tactile-proprioceptive co-occurrences can influence future tactile perception. Furthermore, the authors did not find evidence of a general improvement across the course of the experiment, as performance with hands crossed returned to initial levels of impairment every time posture changed (i.e., from crossed, to uncrossed and back). This detriment in performance following changes in posture might suggest that the brain initializes a fixed, default localization process with every new crossed posture, assuming that touches are located at the anatomical side. Thus, few co-occurrences along the time of an experiment cannot override long-life priors.

A beautiful example of how powerful and long-lasting priors can be when processing touch comes from the Aristotle illusion, first accounted by Aristotle (384–322 B.C.) in the essay ‘‘On dreams’’. In this illusion, a single object is touched with crossed fingers, but strikingly, the individual perceives two rather than one object (Benedetti, 1985). The illusion probably occurs because our brain fails to account for the actual crossed posture of the fingers and processes the sensations arising from the touched object as if the fingers were in their usual uncrossed posture (or, similarly, as if the touch was coming from the anatomical side). Only after months of exposure to this unusual configuration of the fingers, touch takes the real posture into account, and the illusion disappears (Benedetti, 1991). Closely related to this, when two taps are applied in sequence to crossed hands at short intervals, many participants systematically report the first stimuli to occur on the opposite hand (Yamamoto and Kitazawa, 2001; Kóbor et al., 2006; Heed and Azañón, 2014). This can be interpreted as people initially perceiving the location of the touch from the visual side where the hand usually is in space. For instance, for a right-hand touch, the right side of space, which now is occupied by the left hand, would serve as a prior spatial location. Evidence for this comes from visuotactile attention paradigms. When a touch is presented on a crossed hand, quickly followed by a light (<60 ms later), participants are faster in responding to the light in opposite-side (i.e., anatomically congruent) trials than in same-side (i.e., spatially congruent trials). Thus, touches to the left hand, now placed on the right side, facilitate processing of left hemispace visual events and vice versa (see Figures 3A,B; Azañón and Soto-Faraco, 2008a,b; Azañón et al., 2010a). In a similar fashion, a proportion of saccades or reaches directed towards a touch on a crossed limb are initially directed towards the opposite limb, as if they were uncrossed, and then corrected online, several hundred ms later (see Figure 3C; (Groh and Sparks, 1996; Overvliet et al., 2011; see Brandes and Heed, 2015 for reaching trajectory). Finally, it has been shown that disruption of tactile-proprioceptive integration by transcranial magnetic stimulation (TMS) in humans, over the putative right ventral intraparietal cortex, induced participants to underestimate the height of touches delivered to the arm (Azañón et al., 2010b). In this study, participants placed their left arm upright, close to the face, and participants discriminated the location of a touch on the arm, with respect to a touch on the face. The location of the touches on the arm was perceived as coming from a lower position. This could suggest that disruption of tactile-proprioceptive integration by parietal TMS forced touch to rely on an offline proprioceptive representation, in which the arms are represented in their prototypical position, with hands below the face (Azañón et al., 2010b).

In Longo et al. (2010), we introduced the idea that at early stages of tactile processing, and hence, before touch is integrated with an up-to-date proprioceptive representation, the brain assumes for each touch, a stored representation of a canonical posture for that touch. Later, this *a priori* information is overtaken by the actual proprioceptive information or simply weighted less. However, the evidence put forward for this claim (and reviewed in the previous paragraph) does not differentiate between spatial visual priors and canonical postural representations. From a spatial prior perspective, touch is referred in these examples, to the location in visual space where the hand normally is (i.e., the right side of space, for the right hand, or below the face in Azañón et al., 2010b TMS example), without need to account for a particular proprioceptive configuration. From a canonical perspective, however, this effect would be driven by a stored representation of the prototypical layout of the limbs (i.e., a default proprioceptive condition that assumes that the hands are not crossed and placed below the face; see for instance Yamamoto and Kitazawa, 2001).

Regardless of whether these effects are driven by purely visual or by purely proprioceptive priors or a combination of the two, definite and direct evidence for the existence of priors in touch is needed. Note that some direct hypotheses arise from the previous discussion: (1) If tactile stimuli are processed taking into account prior information (in particular, a priori spatial location), one might expect tactile localization biases to occur. (2) If the same skin area is stimulated under different postures, localization biases for that skin area should converge to particular areas of space. Thus, it should be possible to track experimentally these priors touching the same body areas across changes in posture. (3) If tactile stimuli are first processed using a priori information and this a priori information is subsequently adjusted based on the actual spatial location of body parts, then, larger biases should be found at early stages of tactile processing, as compared to later. With regard to possible neural substrates, multimodal neurons with “intermediate” receptive fields in the posterior parietal cortex, and whose activity is gain modulated by the position of the eyes in the orbit, the hand or the head (Pouget et al., 2002; Avillac et al., 2005; Chang and Snyder, 2010) might be able to encode visual priors. Similarly, area PE in the superior parietal lobule (equivalent to BA 5 in the human brain) might be involved in the processing of proprioceptive priors. Some PE neurons in the monkeys react to complex body postures involving several joints (Sakata et al., 1973), and some also respond to tactile stimuli, but only if the limbs and joints are placed in certain positions. Indeed, Sakata and co-workers already suggested that such neurons would be able to encode the spatial position of the touching object relative to the body axis (Sakata et al., 1973).

It is worth noting that the idea of canonical representations of the body is not new. Already in the 1970s, Bromage and Melzack oberved that during the induction of reversible upper and lower limb deafferentation, *via* brachial plexus and epidural anesthesia, participants reported highly stereotyped postures, with arms and legs at their anatomical side, with joints approximately midway through their range of flexion (above the abdomen or lower chest for the arms, and with the legs semiflexed at the hips and (Knees; Melzack and Bromage, 1973; Bromage and Melzack, 1974; see also Gross et al., 1974; Gross and Melzack, 1978). More recent studies have shown that a fully extended finger, wrist, and elbow become a flexed phantom after ischemic anesthesia, though some aspects of the induced phantom sensation change according to the posture held at the time of anesthesia (Inui et al., 2011, 2012a,b). Even though Bromage and Melzack considered these canonical representations outside the frame of tactile processing, the type of proprioceptive priors proposed here might be fundamentally equivalent. Indeed, the authors assumed that this postural archetype may arise by the activity in neural cell assemblies that are developed by earlier sensorimotor activities encountered in a life time, therefore including touch (Melzack and Bromage, 1973). Similarly, a recent study has shown preferential associations between the thumb and the index finger and the relative spatial positions of “top” and “bottom,” suggesting that body parts and spatial locations are stably associated (Romano et al., 2017). In this study, participants were exposed to touches on either the thumb or index fingers. Both hands were placed in front of the body, one on top of the other, with the four stimulated fingers shaping the vertices of an imaginary square and with each homologous fingers (index and thumb) facing each other without touching. In this way, the thumb could be on a relative top position or on a bottom position and vice versa for the index finger. Participants received a single tactile stimulation at one of the four possible locations and were asked to discriminate as quickly as possible whether the top or bottom finger had been touched. The authors found consistent preferential associations between the index finger and the top position and between the thumb and the bottom position, both with and without vision. In this paper, the authors speculated that a canonical postural representation might contribute to somatosensory spatial processing and associate this representation to the fact that for many common grasping actions the index finger is placed in a relatively higher location than the thumb (Romano et al., 2017). This is in agreement with the idea that long-term sensorimotor experience, such as grasping, can create specific functional categories in the brain, which can modulate early stages of somatosensory processing (Shen et al., 2018).

### Examples of Integration of Touch and Online Proprioceptive Information

The idea put forward in this section is that at early stages of tactile processing, possibly before the brain had time to incorporate an online representation of current posture, touch is integrated with (or influenced by) stored representations. This is, however, independent of two facts, i.e., touch necessarily relies on up-to-date proprioceptive information to generate locations in external space, and localization of body parts is tightly linked to visual processing (Limanowski and Blankenburg, 2016). Thus, integration between touch and proprioception for tactile localization often co-occurs with vision (note that other forms of interactions, e.g., with motor commands, are omitted for the sake of brevity; Hermosillo et al., 2011).

The fact that tactile localization is affected by changes in posture (such as hand crossing) is evidence of the integration of touch with online proprioceptive information (Yamamoto and Kitazawa, 2001). There are many other examples in the literature showing effects of posture on somatosensory processing, even when these are visually induced (highlighting the role of vision in body parts localization; Gallace and Spence, 2005; Azañón and Soto-Faraco, 2007; Folegatti et al., 2009). For example, localizing the order of two touches, applied one to each uncrossed hand, becomes easier when the horizontal distance between the two hands increases (Shore et al., 2005). This improvement is observed, even if the separation is not physical, but visually introduced by mirror reflection (Soto-Faraco et al., 2004; Gallace and Spence, 2005). This is the case also for tactile localization with hands crossed (Roberts et al., 2003), which also improves when the separation spans other spatial dimensions (vertical and depth; Azañón et al., 2016a).

Studies on tactile spatial attention further demonstrate the strong interconnection between online postural information and touch (Lakatos and Shepard, 1997; Aglioti et al., 1999; Heed and Röder, 2010). For example, tactile attention to one hand in healthy individuals improves by separating the arms (e.g., Driver and Grossenbacher, 1996; Soto-Faraco et al., 2004). When the task requires switching attention from one hand to the other, then participants’ performance improves by reducing the distance between the arms (Lakatos and Shepard, 1997). Furthermore, when participants discriminate the elevation of a tactile target applied to the index finger or thumb of one hand, there is facilitation from a simultaneous touch on the unattended hand when it is presented in a congruent (e.g., both up) rather than in an incongruent elevation, regardless of the orientation taken by the hand, and therefore the actual finger stimulated (e.g., whether both index fingers are placed on top of the thumbs or a single hand is rotated, and the thumb is on the top of the index finger; Soto-Faraco et al., 2004). Altogether, these results suggest that tactile attention is affected by the posture of the touched body part, given that performance is modulated by the distance and orientation of the body parts despite the somatotopic relationship across the involved skin sites is kept constant in the brain (see also Rinker and Craig, 1994; though see Evans and Craig, 1991; Evans et al., 1992; Röder et al., 2002; Haggard et al., 2006, and Kuroki et al., 2010 for evidence regarding a somatotopic dominance in tactile localization).

Research on patients provides further evidence of the influence of posture in tactile processing. This is the case, for instance, of tactile extinction, already defined in the previous section, or tactile hemineglect, in which tactile stimulation of the contralesional limb (usually the left) is neglected (Vallar, 1997; Driver and Vuilleumier, 2001). The strength of tactile inattention is reduced by the location of the affected body part in space. Thus, some patients improve tactile detection at the contralesional hand when it crosses the midline to the ipsilesional side (Smania and Aglioti, 1995; Moro et al., 2004) or even within the same hemispace when the affected hand crosses the other hand (Aglioti et al., 1999; Moro et al., 2004). Further support comes from patients with extinction anchored to different body parts. In particular, these patients extinguish touches that are presented at the left-most side region of the stimulated body part in external space, say the limb, the hand, or the finger (with respect to their long axis), regardless of the spatial orientation taken by them (e.g. palm up or down; Moscovitch and Behrmann, 1994; Tinazzi et al., 2000; see Medina and Rapp, 2008 for an example in other neurological patients).

Overall, these studies show the impact of postural information in tactile localization. It is important to stress, however, that postural information arises not only from proprioception, but in many instances also from vision. The role of vision in body part localization is evident when a conflict between proprioception and vision is introduced (Rossetti et al., 1995). For instance, in a recent study, Lohmann and Butz (2017) introduced a virtual dissociation of proprioceptive and visual hand position information by combining immersive virtual reality with online motion capturing. They showed that participants unknowingly shifted their hands to compensate for the visual shift. Perhaps the most classical approach to induce visuo-proprioceptive conflict, however, is the rubber hand illusion (RHI, Botvinick and Cohen, 1998). In this classical illusion, participants observe a fake hand being stroked while their real (unseen) hand is synchronously touched. After several seconds of simultaneous stroking, participants tend to perceive the felt tactile sensation as originating from the rubber hand. This usually results in a feeling of ownership and a relocation of the perceived position of the real hand towards the rubber hand (Botvinick and Cohen, 1998; see also Tsakiris and Haggard, 2005). By combining the rubber hand illusion with temporal order judgments with hands crossed, Azañón and Soto-Faraco (2007), found that observing a pair of uncrossed rubber hands reduces the deficit of localizing touches at the hands when crossed. Interestingly, this modulation was mostly observed when visual information about the rubber hands could be attributed to one’s own actions (i.e., when movements of the real hand were mirrored by movements of the rubber hand, in an anatomical fashion), highlighting the role not only of visual information in tactile remapping but also of motor information and the sense of agency.

In summary, we have shown the profound effect that postural information has on tactile processing. However, we have also shown that this is not always the case. Early during development, and in individuals deprived from vision, touch is unaffected by the configuration of the limbs (Röder et al., 2004; Bremner et al., 2008b). Thus, active interaction with the environment and presence of visual inputs seem to modify the way we process and localize touch later in life. As a result of this same interaction, some postural configurations and spatial locations might become associated to particular touches over time, producing what we called canonical postural and spatial priors. We argued that these priors could serve as reference points for localization of tactile events, producing more accurate and faster tactile responses, although biased towards the prior location or proprioceptive configuration. The hypothesis that canonical priors might influence tactile processing is still speculative; however, a growing body of results, some of which have been reviewed here, provides increasing evidence of biases in tactile localization that fit well with the existence of such priors.

## Integration of Tactile Information with Representations of Body Size and Shape

The final form of integration we will discuss is integration of immediate tactile signals with stored representations of body size and shape. Several forms of perception involve referencing sensory signals to models of the body itself. For example, the use of convergence angles for visual depth perception requires that the distance between the two eyes be known (Banks, 1988), while the use of temporal differences when sounds reach the two ears for auditory localization requires that head width be known (Aslin et al., 1983). Other studies have shown, for example, that representation of eye-height affects perception of the passability of doorways (Warren and Whang, 1987; Leyrer et al., 2015), hand size affects the visual size perception (Linkenauger et al., 2010, 2014), and arm length affects the size of peripersonal space (Longo and Lourenco, 2007; Lourenco et al., 2011) and perception of visual distance (Linkenauger et al., 2015). These issues are especially acute in touch, given that the primary receptor surface (i.e., the skin) is physically co-extensive with the body itself.

### The Role of a Body Model in Tactile Distance Perception

A central part of the model of somatoperceptual information processing proposed by Longo et al. (2010) was therefore a stored representation of body size and shape, what they called the *body model*. Stimulation of even single mechanoreceptive afferent fibers in the human median nerve can produce clearly localized tactile sensations (Schady et al., 1983). Imagine, however, that two distinct points on the hand are touched. There is nothing in either of the two resulting signals or their combination that specifies how far apart the two stimuli are. Perceiving the distance between two stimulus locations on opposite sides of the hand effectively reduces the problem of knowing how big one’s hand is. Longo et al. (2010) proposed that this is achieved by combining the location of touch within primary somatotopic maps in somatosensory cortex with the body model.

Evidence in support of this interpretation comes from studies showing that illusions which alter the perceived size or shape of the body produce corresponding changes in the perception of tactile distance. Taylor-Clarke et al. (2004), for example, showed participants a magnified video image of their forearm alongside a minimized image of their hand. Subsequently, the relative perceived distance between two touches was expanded on the forearm and compressed on the hand. Similarly, de Vignemont et al. (2005) explored this issue using the so-called vibrotactile illusion. In the vibrotactile illusion, vibration applied to a muscle tendon produces an illusion of muscle lengthening and a corresponding illusion of proprioceptive limb displacement (Goodwin et al., 1972). Lackner (1988) showed that when this illusion was generated while the affected limb was in continuous contact with another part of the body, illusory changes of experienced body part size could be produced (i.e., the “Pinocchio illusion”). De Vignemont et al. (2005) used this method to produce the illusion that the index finger was longer or shorter than its actual size and showed that such changes affected the perceived distance between touches on the finger, compared to a control skin location (the forehead). Similar results have also been reported in other studies (Bruno and Bertamini, 2010; Tajadura-Jiménez et al., 2012).

Further evidence that higher level representation of the body shapes the perception of tactile distance comes from studies showing that the segmentation of the body into discrete parts produces categorical perception effects, with perceived tactile distances being expanded across joint boundaries (de Vignemont et al., 2009; Le Cornu Knight et al., 2014, 2017; Shen et al., 2018). Similarly, tool use, which can be interpreted as a functional extension of the body (e.g., Maravita and Iriki, 2004), has recently been shown to produce systematic changes in the perception of tactile distance on the arm wielding the tool (Canzoneri et al., 2013; Miller et al., 2014, 2017a,b). Moreover, the nature of these effects is determined by the relation between the tool and the body: a long stick altered touch on the forearm but not the hand, whereas a hand-shaped tool altered touch on the hand but not the forearm (Miller et al., 2014).

### Baseline Distortions of Tactile Distance Perception and the Pixel Model

Intriguingly, even at baseline, there are large misperceptions of tactile distance, which have been investigated since the 19^th^ century. In his classic work, Weber (1996) noticed that as he moved the two points of a compass across his skin it felt like the distance between them increased as they moved from a region of relatively low sensitivity (e.g., the forearm) to a region of higher sensitivity (e.g., the palm of the hand). Subsequent research has replicated these results and found that the perceived distance between touches on the skin has a systematic relation to the relatively sensitivity of different skin regions (Goudge, 1918; Cholewiak, 1999; Taylor-Clarke et al., 2004; Anema et al., 2008; Miller et al., 2016), an effect now known as *Weber’s illusion*.

Interestingly, similar results have also been found comparing the perceived distance between points aligned in different orientations on a single skin surface. For example, Longo and Haggard (2011) found that the perceived distance between touches on the hand dorsum was about 40% larger when the touches were oriented across the width of the hand, than along hand length. Other studies have reported similar results (Longo and Sadibolova, 2013; Calzolari et al., 2017; Longo and Golubova, 2017; Longo, 2017b; Tamè et al., 2017b), and similar anisotropies have been described on a number of skin regions, including the forearm (Green, 1982; Le Cornu Knight et al., 2014), the thigh (Green, 1982), the shin (Stone et al., 2018), and the forehead (Longo et al., 2015a; Fiori and Longo, 2018). Intriguingly, the direction of this effect appears to be the same on all skin regions where anisotropy has been reported, with distances aligned with body width overestimated compared to those aligned with body length or height. However, the magnitude of anisotropy appears to differ systematically across the skin, suggesting that it arises from factors specific to each skin surface rather than a more general perceptual or cognitive bias.

In previous work, we have suggested that such effects may arise from the geometry of the receptive fields (RFs) of neurons in somatosensory cortex, based on what we called the “pixel model” (Longo and Haggard, 2011; Longo, 2017a). The central idea of this model is that tactile RFs in a somatotopic map are treated like the pixels of a two-dimensional spatial image of the body, with distances calculated by counting the number of unstimulated RFs between two activation peaks. Because the RFs representing sensitive skin regions are smaller than those representing less-sensitive regions (Powell and Mountcastle, 1959; Sur et al., 1980), any given stimulus will have more unstimulated RFs between peaks if applied on a sensitive than a less-sensitive surface, potentially accounting for the classic form of Weber’s illusion. Similarly, the RFs of neurons representing the hairy skin of the limbs are generally oval-shaped (rather than circular), with the long axis of the oval aligned with the proximo-distal limb axis (Powell and Mountcastle, 1959; Brooks et al., 1961; Alloway et al., 1989). This anisotropy of RF geometry can potentially account for the perceptual anisotropies described above, given that the spacing between the RFs of adjacent neurons in somatotopic maps is known to be a constant proportion of RF size (Sur et al., 1980). Recent results have been consistent with this model in showing that tactile distance anisotropies can be well characterized by geometrically simple deformations (e.g., stretches) of tactile space (Longo and Golubova, 2017; Fiori and Longo, 2018).

### Tactile Distance Perception and Clinical Disorders of Body Image

A number of recent studies have reported disruption of tactile distance perception in clinical disorders (e.g., Keizer et al., 2011, 2012; Scarpina et al., 2014; Spitoni et al., 2015; Mölbert et al., 2016; Engel and Keizer, 2017). For example, Keizer and colleagues (Keizer et al., 2011, 2012) found that in comparison with healthy controls, patients with anorexia nervosa overestimated tactile distances on both the belly and hand. In a subsequent study, Spitoni et al. (2015) compared tactile distances on the belly and sternum. Patients with anorexia overestimated distances on the belly compared to the sternum, but only when stimuli were aligned with the width of the body and not when they were aligned with body length. This effect is intriguing in that it shows specificity in the distortions of tactile distance perception shown by the patients that mirror their subjective body image (i.e., the fact that they experience their body as fatter than it actually is). Thus, this result provides further evidence for a deep relation between the experience of tactile distance and higher level representation of the body (cf. Longo, 2015).

There is also some evidence that the illusions of tactile distance perception we have described above mirror distortions of body perception in other domains [for review, see (Azañón et al., 2016b; Longo, 2017a)]. For example, studies investigating body representations underlying proprioceptive position sense have reporting similar distortions, with overestimation of hand width relative to length (Longo and Haggard, 2010, 2012a; Ganea and Longo, 2017). Similarly, other studies of the explicit body image have also revealed overestimation of body width, using a range of measures including visual comparison (Shontz, 1969; Longo and Haggard, 2012b), the image marking procedure (Meermann, 1983), the moving caliper procedure (Halmi et al., 1977; Dolan et al., 1987), the adjustable light beam apparatus (Thompson and Thompson, 1986; Dolce et al., 1987), and several others (Bianchi et al., 2008; Fuentes et al., 2013; D’Amour and Harris, 2017). The distortions described above of tactile distance perception thus appear to be just one reflection of a broader perceptual bias to overestimate body width, which appears in many types of task.

## Discussion

In this review, we have explored two aspects of tactile processing that were not considered in the model proposed by Longo et al. (2010), i.e., the integration of touch across the two sides of the body and the use of stored proprioceptive information about the location of touch in space. In addition, we have reviewed recent results concerning the integration of tactile signals with representations of body size and shape since we developed the model.

Regarding the integration of touch across body sides, a large body of evidence, as discussed in the first section, suggests that the integration of tactile signals between the two sides of the body is likely to occur at early stages of tactile processing, i.e., within the primary somatosensory cortex. This line of evidence challenges the textbook account that SI supports only unilateral tactile representations of the contralateral side of the body, whereas structures beyond SI, in particular SII, support bilateral tactile representations. Therefore, in the construction of the somatic percept, the interhemispheric transfer of tactile information occurs very early in time and depends on the spatial and temporal characteristics of the stimuli (Tamè et al., 2012, 2015), the type of task (Tamè et al., 2011, 2014, 2016), as well as the relative position of the parts of the two sides of the body in space (Tamè et al., 2011, 2017c). We propose that such integration occurs in areas 1 and 2 of the primary somatosensory cortex through transcallosal connections as shown by the neuroimaging studies we described (e.g., Tamè et al., 2012, 2015). Following this integrative process, information is then sent to other brain areas within SI (i.e., 3a, 3b), parietal areas, as well as the motor and premotor cortices.

In our previous model (Longo et al., 2010), we proposed that three different types of body representations were required to process touch. Namely, the superficial schema, mediating localization of somatic sensations on the body surface; the model of body size and shape, which was discussed in the last section of this review, and the postural schema, an online and up-to-date proprioceptive representation of the limbs in space. Nonetheless, several considerations converge to support the idea that the processing of touch also involves an offline representation of the most plausible spatial locations for a given touch (Azañón and Soto-Faraco, 2008a; Overvliet et al., 2011) or the most possible configurations of the body in space (Yamamoto and Kitazawa, 2001; Romano et al., 2017). We suggest that these representations or stored information are tightly linked to the postural schema, specially, in the particular case of canonical proprioceptive priors. Minor deviations from this template are maximally informative for comparing current body posture and, in this way, retrieving the up-to-date body schema in a dynamic way. In this hypothetical framework, online sensory information about the tactile stimuli on a body part in a given posture (postural schema) would be combined with information about this offline proprioceptive standard, every time a touch is presented. Consequently, when online information is accurate, both schemata are combined to increase accuracy and speed of tactile processing, as the prior should be seen as the statistical mean for all co-occurrences between touch and this particular body configuration encoded throughout a lifetime.

**Figure 4:**
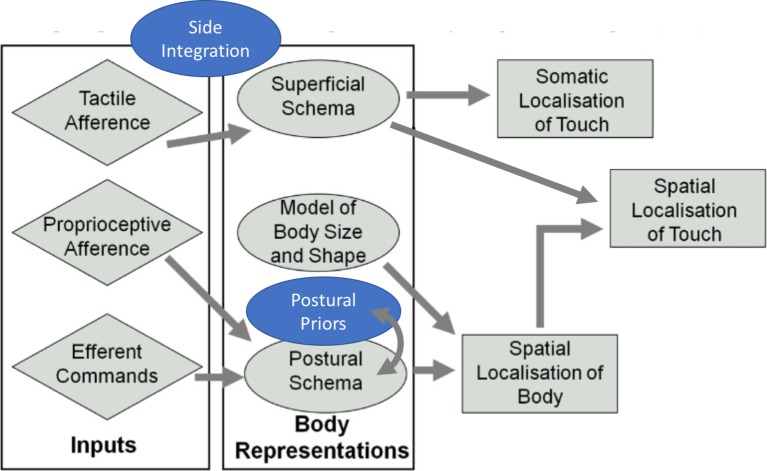
Revised model of somatosensory processing. Addition of the concepts “side integration” and “postural priors” (in blue) to the model initially proposed by Longo et al., 2010. Adapted from Longo et al. (2010). © 2010 by Elsevier. Permission for the use of the image has been obtained from the Elsevier.

Figure 4 shows an updated depiction of Longo et al.’s (2010) model where we have included the notion that touch is necessarily integrated across the two sides of the body. In Figure 4, we suggest that touch is integrated between the two sides of the body before the processing that constructs percepts and experiences of somatic objects and events and of one’s own body (i.e., somatoperception). We have also included a fourth body representation, a canonical prior, to denote the use of priors in the localization of touch. This prior would interact mostly with the postural schema to produce a fast and accurate, though sometimes biased, localization of touch in space.

Taken together, with the inclusion of the concepts of body laterality and prior information, this review provides a more comprehensive conceptualization of tactile processing than our previous model (Longo et al., 2010, 2015a,b). Furthermore, with the revision of a wide range of recent neuropsychological, neuroimaging, and neurophysiological data, we provide evidence that the claims we made 8 years ago are still up-to-date.

## Author Contributions

The three authors contributed equally.

### Conflict of Interest Statement

The authors declare that the research was conducted in the absence of any commercial or financial relationships that could be construed as a potential conflict of interest.
